# COVID‐19 hospitalization with later long COVID in a person with Down syndrome

**DOI:** 10.1002/ccr3.6425

**Published:** 2022-10-07

**Authors:** Mohammad Ashraful Amin, Ishtiakul Islam Khan, Sabrina Nahin, Atia Sharmin Bonna, Sadia Afrin, Mohammad Delwer Hossain Hawlader

**Affiliations:** ^1^ Department of Public Health North South University Dhaka Bangladesh; ^2^ Public Health Professional Development Society (PPDS) Dhaka Bangladesh; ^3^ Department of Physiology Green Life Medical College Hospital Dhaka Bangladesh; ^4^ Public Health Epidemiologist HN & HIV Sector, Save the Children Dhaka Bangladesh

**Keywords:** Bangladesh, COVID‐19, down syndrome, long COVID, risk factor

## Abstract

Viruses that induce pulmonary difficulties and auto‐inflammation are more common in people with Down syndrome. They also have a higher number of comorbidities associated with a worse prognosis than the overall population. Adult patients with acute COVID‐19 are increasingly being diagnosed with Long COVID. However, patients with Down syndrome with later long COVID‐19 are the first example documented in Bangladesh.

## Graphical abstract

COVID‐19: Diagnosis & Hospitalization of a Person with Down Syndrome.

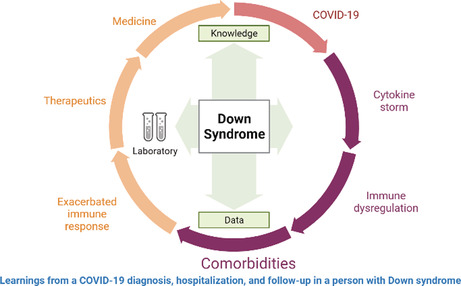

## 1 INTRODUCTION

The most common chromosomal cause of developmental disabilities is Down syndrome (DS), caused by a trisomy of specific gene 21.^1^ It has been linked to several co‐existing health disorders and immunological dysfunction, all of which can influence the disease symptoms and increase the risk of life‐threatening disease from exposure to the emerging severe acute respiratory syndrome coronavirus 2. (SARS‐CoV‐2).^1^ Coronavirus disease (COVID‐19) is primarily a respiratory infection, although it can progress to a severe illness with multi‐organ failure and mortality.^1^ Immune dysregulation in people with Down syndrome makes them more susceptible to viral diseases, while structural airway characteristics make them more susceptible to respiratory infections.^2^, ^3^, ^4^ Respiratory diseases are a leading cause of death in people with Down syndrome.^5^, ^6^ People with Down syndrome appear to be especially vulnerable to coronavirus disease (COVID‐19), with a fourfold increased risk of coronavirus disease (COVID‐19)‐related hospitalization and an estimated three‐fold to 10‐fold more significant risk of coronavirus disease (COVID‐19)‐related fatality.^7^, ^8^, ^9^ Several genetic variants in coordinating immune responses are found on chromosome 21 in Down syndrome, and their amplification causes an increased immune system. Four interferons (IFN) receptors, which function as a sensor and operate for the cytokines interleukin (IL)‐10, IL‐22, and IL‐26, are the primary immunity stabilizers encoded on chromosome 21.^10^ Additionally, people with Down syndrome have immunological and non‐immune cells vulnerable to IFN activation.^11^ This evidence points to interferon‐driven immune dysregulation as a plausible contribution to the formative and clinical cornerstones of Down syndrome, as indicated by steadily increasing interferon alpha and beta receptor subunit 1 (human) (IFNAR1) upregulation in all immune lineages tested by increased expression of the interferon receptors encoded on chromosome 21, as indicated by increased IFNAR1 surface expression in all immune lineages studied. In people with Down syndrome, persistent immunological dysregulation is frequent. As a result, they are more susceptible to infections, particularly bacterial and virus‐related pneumonia.^12^ T‐cell lineages in adults with Down syndrome have been demonstrated in previous research to display significant evidence of heightened activity even in the absence of any evident infections, a trait presumed to be driven by persistent IFN hyperactivity.^13^ As a result, patients with Down syndrome have a strong IFN response, which is vital for elevating antiviral responses and triggering and magnifying the cytokine storm.^3^, ^14^ It is unclear how people with Down syndrome may react to the illness. Espinosa provided solid evidence in a recent analysis that people with trisomy 21 have a higher chance of getting more severe symptoms and have higher hospitalization rates, intensive care admission, secondary bacterial infections, and mortality from SARS‐CoV‐2 infection.^3^ Long COVID, according to the National Institute for Health and Care Excellence in the United Kingdom, includes both continuous symptomatic coronavirus disease (COVID‐19) (with symptoms lasting 4 to 12 weeks) and post‐COVID‐19 syndrome (when they persist beyond 12 weeks in the absence of an alternative diagnosis).^15^ Long COVID indicates that symptoms have persisted in those who have improved from coronavirus disease (COVID‐19). Long COVID can be classified into several types based on the symptoms.^16^ We address adult patients with Down syndrome by reporting the results of COVID‐19, who was hospitalized in a private hospital in Chittagong, Bangladesh. According to our acknowledgement, this is the first case report about the long COVID‐19 syndrome in Down syndrome patients, which would eventually open several doors for research in the future.

## 2 CASE HISTORY

**FIGURE 1:**
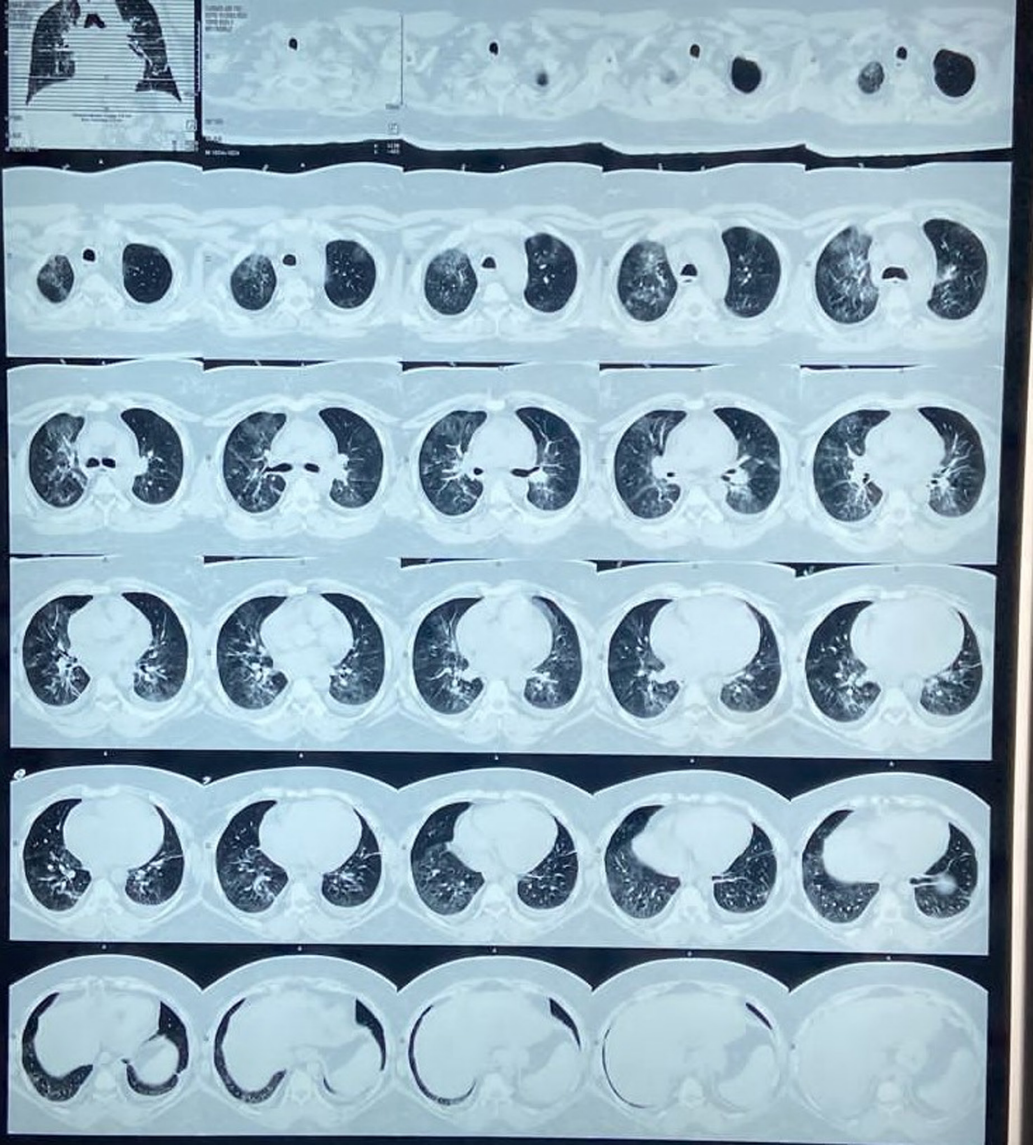
High‐resolution computed tomography (HRCT) of the case

A 42‐year‐old woman with Down Syndrome, a background of hypothyroidism on L‐thyroxine, and diabetes mellitus developed a fever on 10th January 2022, and the next day, she had mild cough and weakness. She had no history of vaccination against COVID‐19. On January 12, 2022, she underwent a coronavirus disease (COVID‐19) test and became positive; the same day, she started tab azythromycin 500 mg once daily, tab montelukast 10 mg once at night and tab paracetamol 500 mg 8 hourly as the physician advised. Her vital were within the standard limit the following week except for temperature. She was admitted to the COVID‐19 department, Feni hospital, on January 21, 2022, with progressively worsening respiratory distress over the last few days and was accompanied by fever, myalgia, and cough. At the time of the patient's admission, the clinical assessment was done, which revealed neurological stability with a Glasgow score of 15/15 and body temperature of 38.2 degrees Celsius with a heart rate of 98 beats per minute, blood pressure (BP) of 130/80 mmHg, peripheral oxygen saturation (SpO2) at ambient air (AA) of 95 percent with Arterial blood gas test (Table 1) and underwent routine and relevant investigation which all were within normal expect C reactive protein (CRP), erythrocyte sedimentation rate (ESR), high‐resolution computed tomography (HRCT) of lung, hemoglobin A1c (HbA1c), random blood sugar (RBS) hemoglobin (Hb) (g/dl) (Table 1). Then, she was referred to Chittagong hospital due to better facilities and got admitted on January 25, 2022. Again, they do, the nasopharyngeal swab polymerase chain reaction (PCR) test for SARS‐CoV‐2 was negative on that day, but a CT scan (computerized tomography) revealed bilateral ground‐glass pneumonia with an estimated 30 percent parenchymal involvement and no sign of pulmonary embolism (Figure 1). She again did the blood test and urine test (Table 1) and started the treatment as per protocol (Table 2), including continuous positive airway pressure (CPAP) due to sudden fall down peripheral oxygen saturation (SpO2) at ambient air (AA) of 88–90 percent. She was transported to the cabin on February 1, 2022, with oxygen delivery at a rate of 2–3 L per minute through a nasal route due to her improved respiratory status. On the February 6, 2022, she was stable, so she was discharged with medication and respiratory exercise. She went to the doctor 3 months later, on May 8, 2022, with complaints of continuous muscular pain in both the upper and lower limbs and joint pain, particularly in the foot. Prior to coronavirus disease (COVID‐19), she had no previous joint or muscle pain history. No morning stiffness, soft tissue swelling, or antalgic gait were also absent. She received standard X‐rays of her upper and lower limbs before being confirmed she had the coronavirus, and nothing noteworthy was seen. The physician prescribed Paracetamols and later NSAIDs for the initial pain.

**TABLE 1:** Investigation parameters of the case

Sl No	Name of the test	Date	Findings	Normal values
01	Blood C/S	02.02.2022	No growth.	
02	High‐resolution computed tomography (HRCT) of Lung	31.01.2022	Bilateral peripheral & central Pneumonitis. Highly suspicious for COVID‐19. CO‐RADS:04. About 30% lungs involved. Other findings: bilateral small pleural effusion.	
03	Procalcitonin	28.01.2022	1.51 ng/ml	less than 0.1 ng/ml
04	Creatinine	24.01.2022	1.2 mg/dl	0.74 to 1.35 mg/dl
		27.01.2022	1.10 mg/dl	
05	Glomerular Filtration Rate (GFR)	27.01.2022	57.0 ml/min/0.73 m3	60 or higher is in the normal range
06	Troponin‐I	27.01.2022	0.64 ng/ml	0 and 0.05 ng/ml
07	C‐reactive protein (CRP)	21.01.2022	126 mg/L	Less than 10 mg/L
		27.01.2022	72 mg/L	
08	D‐Dimer	24.01.2022	3.01 ug/ml	less than 0.50
		27.01.2022	1.65 ug/ml	
09	Alanine transaminase (ALT)	25.01.2022	23 U/L	7 to 55 (U/L)s
		26.01.2022	22 U/L	
10	Serum. Iron	25.01.2022	16 ug/ml	60 to 170 (mcg/dl),
11	25 OH‐Vitamin‐D	24.01.2020	22.5 ng/ml	20 and 40 ng/ml
12	Hemoglobin A1c (HbA1c)	21.01.2022	7.8%	4% and 5.6%
13	Random Blood Sugar (RBS)	21.01.2022	14.75 mmol/L	80 mg/dl and 130 mg/dl
14	Thyroid‐stimulating hormone (TSH)	21.01.2022	6.023 Uiu/ml	0.5 to 5.0 mIU/L
15	Complete Urine Examination (Urine R/M)	21.01.2022	Chemical Examination: Albumin‐Nil Sugar‐Nil Bilirubin‐Nil Ketones‐Positive Nitrate‐Negative Microscopic Examination: RBC‐Nil Pus Cells‐2‐4HPF Epithelial Cells‐0‐1HPF	
16	Febrile Ag/Triple Ag	21.01.2022	Anti‐Rickettsia Ab: OX2‐1:80 OX19‐1:80 OXK‐1:80 Anti‐brucella Ab: Anti B. Abortus‐1.80 Anti B. Melitensis‐1.80 Widal test: TO: 1.80 AO: 1.80 BO: 1.80 TH: 1.80 AH: 1.80 BH: 1.80	1.38 to 187.00 IU/ml for AFP; 1.06 to 315 ng/ml for hCGβ; and 0.25 to 28.5 nmoL/L for uE3
17	S. Electrolytes			
	S. Sodium (Na)‐ mmol/L	02.02.2022	141	135–145 mmol/L
		28.01.2022	140	
		27.01.2022	141	
		26.01.2022	144	
		24.01.2022	136	
	S. Potassium (k)‐ mmol/L	02.02.2022	3.62	3.5 to 5.0 (mmol/L)
		28.01.2022	4.27	
		27.01.2022	4.50	
		26.01.2022	4.33	
		24.01.2022	3.82	
	S. Chloride (Cl)‐ mmol/L	02.02.2022	94	96 to 106 (mmol/L)
		28.01.2022	99	
		27.01.2022	103	
		26.01.2022	102	
		24.01.2022	96	
	S. Calcium (Ca)‐ mmol/L	02.02.2022	0.99	8.6 to 10.3 mmol/L
		28.01.2022	0.74	
		26.01.2022	0.99	
		24.01.2022	1.03	
18	Arterial blood gas test (ABG)	02.02.2022	PH:7.45, pCO2‐46.9, pO2‐48,	
		28.01.2022	PH:7.42, pCO2‐46.4, pO2‐156	
		26.01.2022	PH:7.43, pCO2‐47.4, pO2‐49	
		24.01.2022	PH:7.48, pCO2‐31.9, pO2‐87	
19	Hemoglobin (Hb) (g/dl)	21.01.2022	10.1	14.0–17.5 gm/dl
		27.01.2022	9.4	
20	White blood cells (WBC) (/L)	21.01.2022	6.21 × 10^9^	
		27.01.2022	7.4 × 10^9^	4.5 to 11.0 × 10^9^
21	Platelets	21.01.2022	217 × 10^9^	150 to 400 × 10^9^
		27.01.2022	145 × 10^9^	
22	Neutrophil %	21.01.2022	63	25%–70%
		27.01.2022	86	
23	Erythrocyte sedimentation rate (ESR) (mm)	21.01.2022	92	1–20MCV mm/hr
		27.01.2022	80	
24	mean corpuscular volume (MCV) (FL)	21.01.2022	76.5	80–96 fl
		27.01.2022	78.7	

**TABLE 2:** Treatment

Date	Hospital	Treatment	Status
24.01.2022	Feni Diabetes Hospital	Inj. Meropenem (1 gm) Inj. Dexamethasone (5 mg) Inj. Enoxaparin sodium (60 mg) Inj. Actrapid 100 IU Inj. Remdesivir Inj. Levofloxacin (500 mg) Tab. Paracetamol (500 mg) Tab. Baricent 2 mg Tab. Levothyroxine 50 micrograms Tab. Cholecalciferol (Vitamin D3) Tab. Montelukast (10 mg) Inj. Omeprazole (40 mg) Tab. Acetylcysteine (600 mg) Inh. Salbutamol and Ipratropium Bromide Tab. Pirfenidone (267 mg)	Refer to CMCH
30.01.2022	Medical Centre Hospital	Inj. Meropenem (1 gm) Inj. Dexamethasone (5 mg) Inj. Vitamin complex Inj. Enoxaparin sodium (60 mg) Inj. Levofloxacin (500 mg) Inj. Actrapid 100 IU Tab. Baricent 2 mg Tab. Acetylcysteine (600 mg) Tab. Levothyroxine 50 micrograms Tab. Omeprazole (40 mg) Tab. Montelukast (10 mg) 2% Miconazole gel	Ongoing treatment
02.02.2022	Medical Centre Hospital	Cap. Denvar (400 mg) Inj. Dexamethasone (5 mg) Inj. Enoxaparin sodium (60 mg) Inj. Actrapid 100 IU Tab. Paracetamol (500 mg) Tab. Baricent 2 mg Tab. Levothyroxine 50 micrograms Tab. Cholecalciferol (Vitamin D3) Tab. Montelukast (10 mg) Inj. Omeprazole (40 mg) Tab. Acetylcysteine (600 mg) Inh. Salbutamol and Ipratropium Bromide Tab. Pirfenidone (267 mg) Tab. Clonazepam 0.5 mg Tab. Quetiapine 50 mg	Ongoing treatment and later discharge.

## 3 DISCUSSION

To the best of our knowledge, this may be the first reported in Bangladesh with this combination of Down syndrome and coronavirus disease (COVID‐19) patients.^17^ People with intellectual disabilities are the unique issues resulting from the coronavirus disease (COVID‐19) epidemic.^17^ Patients with Down syndrome, who commonly suffer from intellectual impairment.^18^ With a prevalence of about 1 in 1000 live births, Down syndrome seems to be the most prevalent chromosomal defect in people around the globe. The literature considers an estimated prevalence of Down syndrome of around 0.125% in Bangladesh.^19^ Individuals with Down syndrome have unique socio‐demographic potential risks for coronavirus disease (COVID‐19). They are more likely to have complications such as obesity, diabetes, congenital heart disease, and respiratory disorders linked to a worse coronavirus disease (COVID‐19) outcome in the overall population.^20^ Furthermore, The production of cytokines that are more involved in triggering a prothrombotic procoagulant reaction^21^ and Down syndrome may be an established risk factor for thromboembolic illness and an increased risk of cardiovascular episodes.^22^, ^23^ There is a higher incidence of respiratory infections, immunological dysfunction, systemic inflammation, early aging, and complications linked with coronavirus disease (COVID‐19) risk, contributing to poor patient outcomes. However, it is uncertain whether they are more prone to SARS‐CoV‐2 infection.^3^

The transmembrane serine protease 2 (TMPRSS2)‐ (*602060)* gene is found on chromosome *21q22.3*, suggesting that it may be overexpressed in people with Down syndrome. The protein produced by this gene is related to the increase in TMPRSS2 receptors at the molecular level. As a result, it is reasonable to believe that this contribution may account for some of these people's more severe COVID‐19 cases. Studies in Down syndrome patients can help researchers learn more about the processes behind the infectious process in COVID‐19, which will help them better understand and prioritize treatments for severe instances in the overall population.^24^

This case demonstrates the need for more clinical and scientific research into the genetic susceptibilities that influence the severity of COVID‐19 and SARS‐CoV‐2‐related problems. While there is an apparent dearth of systematic epidemiological data on coronavirus disease (COVID‐19) in Down syndrome patients, we want to draw attention to this hyperinflammatory and life‐threatening presentation in adults with Down syndrome to ensure the early clinical diagnosis of comparable cases in the ongoing SARS‐CoV‐2 pandemic.

In one research, hospital individuals with Down syndrome and coronavirus disease (COVID‐19) had a relative risk of mortality of 2.9 compared to controls.^25^ Since the H1N1 outbreak in Mexico in 2009, the chances of intubation and mortality were eightfold and 335‐fold higher, respectively, for individuals with Down syndrome than for others.^26^ One research of 12 people with Down syndrome and coronavirus disease (COVID‐19) revealed that those admitted with COVID‐19 had a worse illness than their age‐matched counterparts.^27^ In these two investigations, people with Down syndrome are identified as a high‐risk population for severe COVID‐19 with a poor prognosis. Difficulty breathing, fever, coughing, and muscle fatigue were the most common signs and symptoms of coronavirus disease (COVID‐19) in patients with Down syndrome.^4^ This case report supports this observation. On the other side, patients with Down syndrome had a more severe condition than controls, with a higher risk of sepsis and the need for mechanical breathing, according to a prior study.^27^ It is possible that in the first wave of the pandemic, people with Down syndrome were hospitalized later due to diagnostic delays, resulting in even worse clinical outcomes. This tendency, however, has not been seen in the overall population who have been treated for SARS‐CoV‐2 pneumonia.^28^, ^29^ This patient was diagnosed as soon as her symptoms began, and she received rapid treatment for her problem and additional investigations. Consequently, this patient was discharged from the hospital in a relatively stable condition. In coronavirus disease (COVID‐19) individuals with Down syndrome, the main complication for inpatient and death was age, which is in line with evidence from the general population as published in previous Coronavirus Clinical Characterization Consortium (ISARIC4C) survey data.^25^ Significantly, we noticed an elevated death rate starting at 40, much younger than the entire populace. Many indications of accelerated aging have been extensively observed in people with Down syndrome.^30^ In our case, the patient's age of 42 was a risk factor during admission into the hospital for coronavirus disease (COVID‐19).

Her BMI was within the usual range in our circumstance. In adults with Down syndrome, calculating BMI should be done once a year to check for weight gain and obesity. Adults should follow the United States Preventive Services Task Force (USPSTF) recommendation for behavioral weight loss strategies to minimize obesity‐related morbidity and mortality.^31^ Given the increased risk of cardioembolic stroke in individuals with Down syndrome who have a history of congenital heart disease, a cardiologist should do a periodic cardiac assessment and develop a surveillance plan.^31^ There were no documented cardiac complications or anomalies in our patient.

Long COVID refers to the presence of numerous symptoms weeks or months after contracting SARS‐CoV‐2 infection, regardless of viral state. It is also known as “post‐COVID syndrome.” It might be either persistent or relapsing and remitting. There may be a persistence of one or more acute COVID symptoms and the emergence of new symptoms. Most persons with post‐COVID syndrome test positive for polymerase chain reaction (PCR), showing that their microbiological health has improved.^16^ Post‐COVID or long COVID can be separated into two stages depending on the duration of symptoms. COVID is classified as post‐acute when symptoms last longer than 3 weeks but less than 12 weeks, and chronic when symptoms last longer than 12 weeks.^16^ Our patient suffered from muscle pain and joint pain for more than 12 weeks.

Azithromycin has direct and indirect antiviral activity in bronchial epithelial cells.^32^ The benefit–risk profile of azithromycin medicines in COVID‐19 patients to prevent bacterial superinfection is still being studied.^33^ According to studies, doctors should consider using a medicine called baricitinib, which disrupts a signaling pathway necessary for the interferon response. His group demonstrated that it reduced otherwise‐lethal immunological hypersensitivity in mice with trisomy 21 in a study published last month in cell reports.^34^ This shows that baricitinib might assist coronavirus disease (COVID‐19) patients with Down syndrome in regulation an out‐of‐control immune response. The Food and Drug Administration has approved baricitinib in proper selection with remdesivir for emergency use in COVID‐19 patients hospitalized and critically unwell.^34^ In addition to antibiotics and other drugs, our patient received remdesivir for her symptoms. In that case, Baricitinib cannot be used for trial due to its new approval and limited availability, and she recovered without any significant issues without it.

Limitation, we solely focused on hospital admissions; outcomes in the specific community (including asymptomatic and mild COVID‐19 cases) might vary. Despite being seen by a doctor every year and having her whole medical history thoroughly gathered, she had not yet had any testing for connective tissue disorders, which can emerge at any time. As a result, this may be additional limitation. The main goal of the patient's musculoskeletal evaluation was to find any signs of inflammatory arthritis. Since documentation on a large number of musculoskeletal anomalies was only obtained from medical history, the prevalence of some conditions may be understated.

It is time to consider COVID‐19 syndrome in general and Down syndrome in particular as a side effect of long coronavirus disease (COVID‐19). If we did not start planning for this right away, we could manage musculoskeletal disorders, especially in patients with Down syndrome who struggle to accurately pinpoint the location and degree of their discomfort.

## 4 CONCLUSION

Our outcomes suggest that patients with Down syndrome should pay special attention to COVID‐19 early identification, management, and prevention since they are at a higher risk of hospitalization‐related complications during the coronavirus disease (COVID‐19) outbreak. Individuals with Down syndrome are high‐risk groups for significant COVID‐19 infection and should get the vaccine quickly. Additionally, if they become hospitalized due to the disease, they should receive more rigorous support and care. Effective strategy from both family members and local practitioners is required for individuals with Down syndrome to adhere to the necessary guidelines. To summarize, patients with Down syndrome have multiple risk factors for respiratory infections and poor outcomes due to many comorbidities, anatomical changes in the upper respiratory tract, and immunological dysregulation. Individuals with Down syndrome are among the priority candidates for early immunosuppression, current antiviral treatments, and, once accessible, the SARS‐CoV‐2 vaccine. The widely accepted health monitoring standards must be updated for long coronavirus disease (COVID‐19) to include an annual musculoskeletal examination for all children and adults with Down syndrome.

## AUTHOR CONTRIBUTIONS

The article's first draft was written by MAA and MDH. MAA, IIK, SN, ASB, SA, and SD contributed to the literature review and manuscript preparation. All authors contributed to the final version by critically reviewing and editing drafts.

## FUNDING INFORMATION

This research did not receive any specific grant from funding agencies in the public, commercial, or not‐for‐profit sectors.

## CONFLICT OF INTEREST

The authors declare that they have no competing financial interests or personal relationships that could have appeared to influence the work reported in this paper.

## ETHICAL APPROVAL

The article is about a case study. As a result, our Ethics Committee's consent was not required.

## CONSENT

The patient's parents had written informed consent taken for publishing this case report because of patient developmental delay and images also were acquired.

## Data Availability

Data can be shared based on the reader's reasonable request and priority base and some restrictions will apply.
